# Dataset on fabrication of an improved L-lactate biosensor based on lactate oxidase/cMWCNT/CuNPs/PANI modified PG electrode

**DOI:** 10.1016/j.dib.2018.02.010

**Published:** 2018-02-12

**Authors:** Kusum Dagar, C.S. Pundir

**Affiliations:** Department of Biochemistry, Maharshi Dayanand University, Rohtak, India

**Keywords:** L-Lactate oxidase, Nanomaterials, Lactic acid, Plasma, Pencil graphite electrode, Covalent binding

## Abstract

The data shown in this article are based on the original research article entitled “An improved amperometric L-lactate biosensor based on covalent immobilization of microbial lactate oxidase onto carboxylated multiwalled carbon nanotubes/copper nanoparticles/ polyaniline modified pencil graphite electrode” (Dagar and Pundir, 2017) [1]. This article explains the fabrication of an amperometric L-lactate biosensor based on microbial lactate oxidase (LOx) covalent immobilization onto nanomatrix [(carboxylated multiwalled carbon nanotubes (cMWCNT)/copper nanoparticles (CuNPs)/polyaniline (PANI) hybrid film/pencil graphite electrode (PGE)]. The dataset based on this article is made publically available for critical analysis. The whole data is supplied in the research article instead of repository. The data in the article is not related to any already published article.

**Specifications Table**

**Table:** 

Subject area	*Analyical Biochemistry*
More specific subject area	*Nanomaterials based Biosensor*
Type of data	*Manuscript, Tables, Figures*
How data was acquired	*By characterization of CuNPs: TEM, XRD, UV spectrophotometer (Dynamica HALO DB-20, UK), enzyme electrode by SEM (ZeissEV040), FTIR (Thermo Scientific iS10, USA), EIS, CV potentiostat-galvanostat (Eco-Chemie The Netherland, Autolab, model: AUT 83785,), measuring biosensor response (In current) at different pH, temp, concentrations of lactate, studying analytical recovery of added lactic acid in plasma determined, precision for lactate determination in plasma, correlating with standard enzymic colorimetric method for plasma lactate and quantification of lactate in plasma of apparently healthy and lactoacidosis patients, dairy products, orange juice and alcoholic beverages.*
Data format	*Analyzed data*
Experimental factors	*Dilution of some of the biological samples in DW.*
Experimental features	An improved amperometric lactate biosensor was constructed by immobilizing LOx covalently onto hybrid film of cMWCNT/CuNPs/PANI electrodeposited onto PG electrode. The improved biosensor showed a very rapid response (5 s), with a lower detection limit (0.25 µM) and broader linear range (1 µM to 2500 µM), good reproducibility and higher storage stability (140 days). Thus the use of cMWCNT/CuNPs/PANI hybrid film has improved the analytical performance of a lactate biosensor and could also be used for the improvement of other biosensors.
Data source location	Blood plasma samples from local hospital of Pandit Bhagwat Dayal Sharma Postgraduate Institute of Medical Sciences, commercially available milk products: Buffalo milk and curd from buffalo milk, cheese and yogurt (Brand name: Amul), red wines prepared from purple grapes (Brand name: Sauvignon Blanc, Cabernet Blend, Merlot, Nine Hills Chenin Blenc and Sula Chenin Blanc and beer; Brand name: Orangeboom, Hoegarden, Tsingtao, Heineken and Tuborg) from local market were used.
Data accessibility	The data are available in this article

TEM = Transmission electron microscopy, SEM = Scanning electron microscopy, XRD = X-ray diffraction, FTIR = Fourier transform infrared spectroscopy, EIS = Electrochemical impedance spectroscopy, CV = Cyclic Voltammetry.

**Value of the data**

• The present work describes the construction of an amperometric lactate biosensor with improved response time, limit of detection, working range and storage stability.
• Biosensor can be used to measure lactate in plasma with high accuracy and specificity, which is an excellent indirect marker of cellular fatigue and critical in the patients suffering from lactoacidosis.
• The biosensor could be miniaturized into commercial model/portable model and thus could be used at the bedside of the patient.
• The biosensor showed better analytical performances than the earlier reported biosensor [2], [3], [4].
• This data allows other researchers to fabricate another biosensor on the same nanomatrix with some modifications as the nanomatrix provided excellent results in the present biosensor.

## 1 Data

**Fig. 1:**
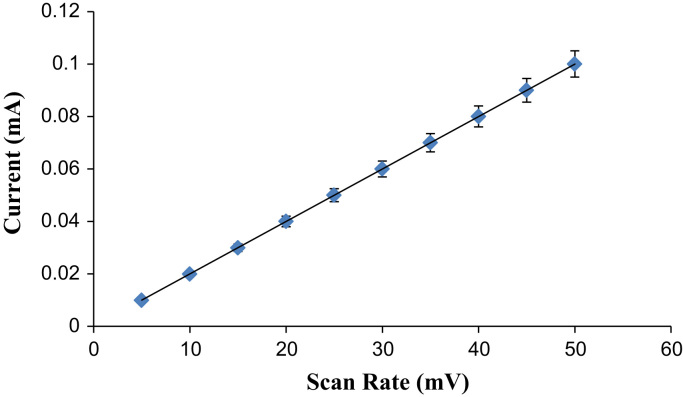
Effect of scan rate to the response of Lactate Biosensor.

**Fig. 2:**
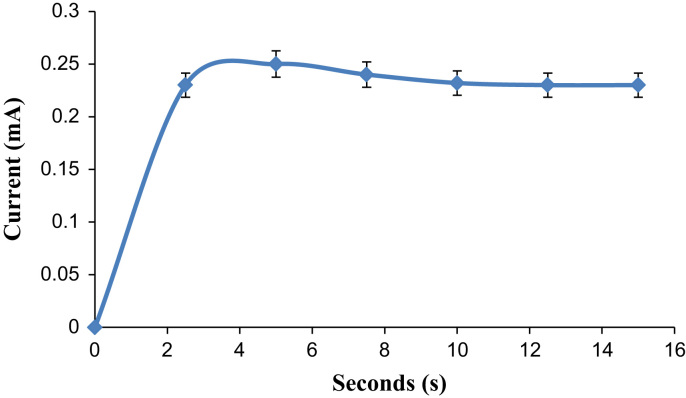
Effect of response time on Lactate Biosensor.

**Fig. 3:**
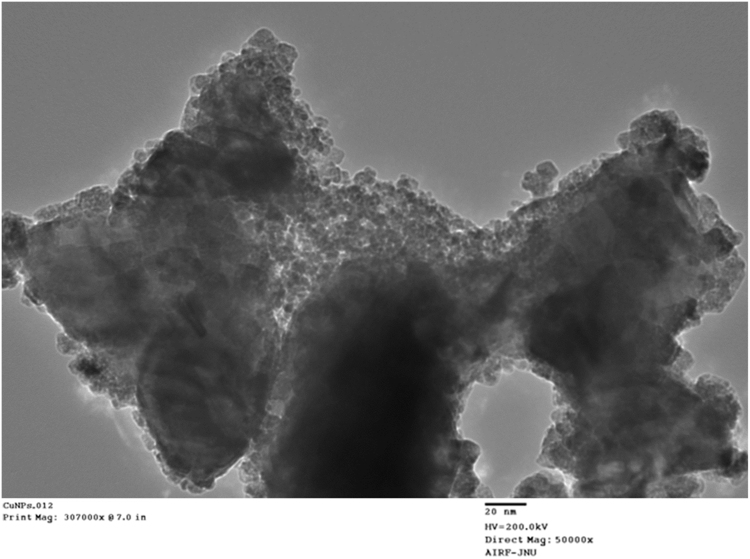
Transmission electron microscopic (TEM) images of CuNPs.

As mentioned in the article, the biosensor exhibited better analytical performances as compared to the other lactate biosensors. The some analytical characteristics are also described in this dataset such as optimum scan rate and response time. The designed biosensor worked at optimum scan rate of 20 mV/s and with a rapid response time (5 s). Fig. 1, Fig. 2, Fig. 3 represent the effect of scan rate, incubation time on response of L-lactate biosensor and TEM images of copper nanoparticles.

## 2 Experimental design and materials and methods

### 2.1 Experimental design

1. Preparation and characterization of CuNPs.
2. Electrodeposition of cMWCNT/CuNPs/PANI onto PG electrode.
3. Immobilization of LOx onto cMWCNT/CuNPs/PANI onto PG electrode.
4. Physico-chemical characterization of enzyme electrodes at different stages of its construction.
5. Construction and testing of amperometric L-lactate biosensor.
6. Optimization of L-lactate biosensor.
7. Evaluation of L-lactate biosensors.
8. Application of L-lactate biosensor in determination of L-lactate in blood plasma in healthy persons and patients suffering from lactoacidosis.

### 2.2 Materials and methods

L-Lactate oxidase (LOx from Pediococcus species), (L-0638, LOx 100 units/mg) from Sigma Aldrich USA and carboxylated multi-walled carbon nanotubes (cMWCNTS) from Intelligent Materials Pvt. Ltd. Panchkula, tetraethylorthosilicate (TEOS) from Fluka Mumbai were used. All other chemicals (AR grade) were from SRL Mumbai. Double distilled water (DW) was used during the experimental studies. Blood plasma samples were collected from hospital of local Pandit Bhagwat Dayal Sharma Postgraduate Institute of Medical Sciences. Commercially available milk products, various wines prepared from purple grapes with brand name as Sauvignon Blanc, Cabernet Blend, Merlot, Nine Hills Chenin Blenc and Sula Chenin Blanc and beer (Brand name: Orangeboom, Hoegarden, Tsingtao, Heineken and Tuborg) were purchased from local market.

CuNPs were prepared by chemical reduction method [1], enzyme electrode was fabricated by immobilizing LOx onto cMWCNT/CuNPs/PANI modified PG electrode by EDC/NHS chemistry and lactate biosensor was constructed by connecting LO electrodet with Ag/AgCl electrode and Pt wire through potentiostat. Biosensor's response was measured amperometrically. Biosensor was applied for determination of lactate in biological materials using standard curve between lactate concentration vs. current in mA under optimum working conditions (Table 1, Table 2).

**Table 1:** Physico- Chemical properties of CuNPs.

**SN.**	**Technique used**	**Value of technique**
1.	TEM : Size	4.28, 6.35, 7.05, 8.07 nm
	Shape	Spherical
2.	UV and visible spectra: Peak at	650 nm
3.	FTIR Spectra: Peaks at	(i) 2922.39 cm^−1^ (ii) 1028.20 cm^−1^ to 1056.90 cm^−1^ (iii) 1583.52 and 1047.88 cm^−1^
4.	EIS Spectra: Rct value	480 Ω, 245 Ω, 320 Ω

**Table 2:** Analytic properties/characteristics of L-lactate biosensor based on LOx/cMWCNT/CuNPs/PANI/PG electrode.

**SN.**	**Characteristics**	**Value**
1.	Working Potential	20 mV
2.	Optimum pH	pH 8.0
3.	Optimum temp.	37 °C
4.	Response time	5 s
5.	Working range/linearity	1–2500 µM
6.	Limit of Detection	95.5%
7.	Analytic recovery	6.24%
8.	Precision: Within and between batch CV	4.19%
9.	Correlation with standard method	R^2^=0.97
10.	Reusability	180 times
11.	Storage stability at 4 °C	140 days
12.	Interference by metabolites	Nil
