# Supplementary material for: Dataset on fabrication of an improved L-lactate biosensor based on lactate oxidase/cMWCNT/CuNPs/PANI modified PG electrode
Source: Data Brief. 2018 Feb 12;17:1163–7. doi: 10.1016/j.dib.2018.02.010 (PMC5988403; doi:10.1016/j.dib.2018.02.010)
Supplement: Supplementary file 1 — Supplementary material [file mmc1.docx]

**DEPARTMENT OF BIOCHEMISTRY**

**MAHARSHI DAYANAND UNIVERSITY**

**ROHTAK – 124 001, Haryana (INDIA)**

To

Dr. Marina Ostankovitch, PhD

Senior Scientific Editor, Data in Brief

Enzyme and Microbial Technology

**Sub:** **Declaration of no conflicts of interest** regarding the revised DIB of MS# DIB-D-16-00830R4 of paper entitled ***“***Dataset on fabrication of an improved L-lactate biosensor based on lactate oxidase/cMWCNT/CuNPs/PANI modified PG electrode**”** by Kusum Dagar and C.S. Pundir*

Dear Dr. Marina Ostankovitch

I hereby declared that there is no conflict of interest among the authors, regarding the revision of DIB-D-16-00830R4 of our paper entitled ***“***Dataset on fabrication of an improved L-lactate biosensor based on lactate oxidase/cMWCNT/CuNPs/PANI modified PG electrode**”** by Kusum Dagar and C.S. Pundir*. The manuscript in the present form has been approved by the co-author.

Yours sincerely,

**Emeritus Prof. C.S. Pundir, FRSC**

Dept. of Biochemistry, M.D.University, Rohtak-124001, Haryana, India

Corresponding Author

E-mail: [pundircs@rediffmail.com](mailto:pundircs@rediffmail.com).

Dated: 29Jan,2018
